# Downregulation of LRRC8A protects human ovarian and alveolar carcinoma cells against Cisplatin-induced expression of p53, MDM2, p21^Waf1/Cip1^, and Caspase-9/-3 activation

**DOI:** 10.1152/ajpcell.00256.2015

**Published:** 2016-03-16

**Authors:** Belinda Halling Sørensen, Dorthe Nielsen, Unnur Arna Thorsteinsdottir, Else Kay Hoffmann, Ian Henry Lambert

**Affiliations:** ^1^Department of Biology, Section of Cell Biology and Physiology, The August Krogh Building, University of Copenhagen, Copenhagen, Denmark

**Keywords:** platinum drugs, multidrug resistance, organic anion channels, transcription factors, taurine, apoptosis

## Abstract

The leucine-rich repeat containing 8A (LRRC8A) protein is an essential component of the volume-sensitive organic anion channel (VSOAC), and using pharmacological anion channel inhibitors (NS3728, DIDS) and LRRC8A siRNA we have investigated its role in development of Cisplatin resistance in human ovarian (A2780) and alveolar (A549) carcinoma cells. In Cisplatin-sensitive cells Cisplatin treatment increases p53-protein level as well as downstream signaling, e.g., expression of p21^Waf1/Cip1^, Bax, Noxa, MDM2, and activation of Caspase-9/-3. In contrast, Cisplatin-resistant cells do not enter apoptosis, i.e., their p53 and downstream signaling are reduced and caspase activity unaltered following Cisplatin exposure. Reduced LRRC8A expression and VSOAC activity are previously shown to correlate with Cisplatin resistance, and here we demonstrate that pharmacological inhibition and transient knockdown of LRRC8A reduce the protein level of p53, MDM2, and p21^Waf1/Cip1^ as well as Caspase-9/-3 activation in Cisplatin-sensitive cells. Cisplatin resistance is accompanied by reduction in total LRRC8A expression (A2780) or LRRC8A expression in the plasma membrane (A549). Activation of Caspase-3 dependent apoptosis by TNFα-exposure or hyperosmotic cell shrinkage is almost unaffected by pharmacological anion channel inhibition. Our data indicate *1*) that expression/activity of LRRC8A is essential for Cisplatin-induced increase in p53 protein level and its downstream signaling, i.e., Caspase-9/-3 activation, expression of p21^Waf1/Cip1^ and MDM2; and *2*) that downregulation of LRRC8A-dependent osmolyte transporters contributes to acquirement of Cisplatin resistance in ovarian and lung carcinoma cells. Activation of LRRC8A-containing channels is upstream to apoptotic volume decrease as hypertonic cell shrinkage induces apoptosis independent of the presence of LRRC8A.

under physiological and pathophysiological conditions and following chemotherapeutic treatment, outdated/damaged cells become eliminated by apoptosis. This cell death process is characterized by sequential net loss of osmolytes (chloride, potassium, amino acids), cell shrinkage [termed apoptotic volume decrease (AVD)], DNA fragmentation, cleavage/activation of executor caspases, and formation of apoptotic bodies, which in our human body is removed by blood-circulating macrophages (8). Platinum derivatives have for a long time been recognized for their clinical antitumor activity and are commonly used in chemotherapeutic treatment (3). Cisplatin [*cis*-diamine dichloroplatinium (II)] was the first member of a class of platinum-based anticancer drugs, which also includes carboplatin and oxaliplatin. Once administrated to the patients' blood circulation, Cisplatin accumulates in the cell cytosol/nucleus, where an aquation reaction causes the formation of a highly reactive aqualigand (3). Binding of platinum (II) to the nucleobases causes the formation of DNA adducts and interstrand cross-links that might lead to DNA lesions and trigger the intrinsic cell death pathway. The latter involves the ataxia telangiectasia mutated and the RAD3-related protein kinases (ATR/ATM) as well as the transcription factor p53, and culminates in the activation of apoptosis (12, 24) and/or cell cycle arrest (10). Human p53 is described as the “guardian of the genome” due to its ability to control the expression of several genes and miRNAs affecting cellular processes, e.g., proliferation, DNA repair, apoptosis, autophagy, metabolism, and migration (40). The protein level and transcriptional activity of p53 is under normal physiological conditions kept at low levels by the E3 ubiquitin ligase MDM2 which binds to p53's transactivation domain and targets p53 toward proteasomal degradation (4, 35). DNA damage mediated by Cisplatin treatment leads to a rise in p53 protein stability through ATR/ATM-mediated phosphorylation (35) and subsequently affects cell fate through increased transcription of *1*) the cyclin-dependent kinase inhibitor p21, which blocks cyclin-dependent kinase 1 and 2 (CDK-1 and -2) and causes G1/S and G2/M cell cycle arrest; and/or *2*) PUMA (p53-upregulated modulator of apoptosis) and Noxa (PMAIP1, phorbol-12-myristate-13-acetate-induced protein 1), which via prevention of the interaction between proapoptotic members of the Bcl-2 family (Bax/Bak) and anti-apoptotic proteins stabilizes the proapoptotic Bcl-2 family proteins. The latter oligomerize to form pores in the mitochondrial membrane and facilitate cytochrome c and SMAC (second mitochondria-derived activator of caspases) release from the mitochondria into the cytosol. Once released cytochrome c promotes Caspase-9 activation on a cytosolic scaffold protein (APAF-1, apoptotic protease activating factor 1) and SMAC concomitantly lifts the effect of the caspase inhibitor XIAP (X-linked inhibitor of caspases). Caspase-9 subsequently activates the effector Caspases-3, -6 and -7, i.e., the execution of apoptosis (8).

Despite that Cisplatin has been proven to be a highly effective chemotherapeutic agent, cancer cells tend to develop resistance to the drug whereupon its efficacy vanishes with time and cancer treatment fails. Drug resistance can be an innate property (intrinsic resistance) due to permanent genetic changes/cell differentiation or an acquired property due to adaptations by the tumor cells (extrinsic resistance) (24). The mechanism of platinum(II) resistance involves direct changes in *1*) drug transport systems resulting in a reduced drug uptake via the copper transporter 1 (CTR1) and increased drug efflux via the copper transporting ATPases (ATP7A and ATP7B); *2*) an enhanced drug detoxification due to elevated levels of intracellular scavengers as glutathione and/or metallothio-proteins; *3*) changes in DNA repair involving increased nucleotide excision, interstrand crosslink and/or less mismatch repair; *4*) changes in the tolerance toward DNA damage; or *5*) changes in the apoptotic signal transduction pathways (12, 24, 25, 32, 46). However, within recent years, it has been found that acquirement of chemotherapeutic resistance in addition to the changes above also implies a modulated activity of volume-sensitive transporters/channels for ions and organic osmolytes, which limits AVD and ultimately impairs the initiation of apoptosis (17). Using wild-type mouse Ehrlich ascites tumor cells (EATC-WT), Hoffmann and coworkers (37) demonstrated that AVD is a three-step process which reflects a balance between the activities of pro- and antiapoptotic transporters for ions and organic osmolytes. The first phase (termed AVD_1_) reflects net loss of KCl and amino acids through activation of separate, proapoptotic channels (VRAC ≈ volume-regulated anion channel; VSOAC ≈ volume-sensitive organic anion channel; TASK-2 ≈ KCNK5 ≈ potassium channel subfamily K member 5); The second transient phase (termed AVD_T_) reflects net uptake of extracellular NaCl via antiapoptotic exchange systems (NHE1: Na^+^/H^+^ exchanger) or cotransporters (NKCC1: Na^+^/K^+^/2Cl^−^); The third phase (termed AVD_2_) implies massive loss of osmolytes which reflects the inability of the antiapoptotic systems to compensate osmolyte loss via proapoptotic transporters (37). The activity of the proapoptotic channels is often seen downregulated or even absent in drug-resistant cancer cells, e.g., human epidermoid carcinoma KCP-4 cells, human lung epithelial A549 cells (A549/CDDP), EATC (EATC-MDR), human leukemia cells (HL60/AR), and human ovarian A2780 (A2780CisR) (13, 30, 33, 37, 44). Furthermore, pharmacological inhibition by administration of more or less specific anion-channel blockers are reported to cause drug resistance in, e.g., human epidermoid KB-3-1, human non-small-cell lung A549, erythroleukemia K562, HL60 promyelocytic leukemia, and MG-63 osteosarcoma cells, as well as EATC (5, 13, 30, 33, 37).

The volume-sensitive channels for anions and organic osmolytes (VSOAC/VRAC) are ubiquitously expressed in mammalian cells (18) and in April 2014 two separate groups independently identified members of the leucine-rich repeat containing 8 (LRRC8) family as being essential compounds/regulators of VRAC and VSOAC (39, 45, 49). The LRRC8 family includes five members (LRRC8A to LRRC8E), and it has been suggested that the functional channel is formed by 6 proteins from the LRRC8 family (1) and that a shift in stoichiometry between LRRC8 members affects channel activity/selectivity (44). In this regard, it was demonstrated that the swelling-induced Cl^−^ conductance via VRAC is mediated by LRRC8A, LRRC8C, and LRRC8E (49), whereas swelling-induced taurine efflux via VSOAC relies on LRRC8A and LRRC8D (36). LRRC8A has been identified as an essential component for both VRAC and VSOAC activities, as reduced expression of LRRC8A correlates with a reduced ability to regulate cell volume in human ovary (A2780), colon (HCT116), cervical (HeLa), and embryonic kidney (HEK) cells, as well as in T-lymphocytes (39, 44, 49). Furthermore, LRRC8A expression has been demonstrated to correlate with the swelling-induced release of the organic osmolyte taurine from, e.g., HeLa (39), HCT116 (49), human ovarian A2780 cancer cells (44) and primary rat astrocytes (20). Finally, LRRC8A is also required for ATP-induced release of glutamate and taurine from nonswollen rat astrocytes (20). A role of LRRC8 family members in cisplatin transport is recently reviewed by Jentsch and coworkers (22).

With respect to acquirement of Cisplatin resistance, data from our group show that LRRC8A is markedly downregulated in Cisplatin-resistant A2780 (A2780CisR) cancer cells compared with the Cisplatin-sensitive parental A2780WT, and that this downregulation correlates with an absent swelling-induced taurine efflux (44). Furthermore, we found that 18 h Cisplatin exposure resulted in a 2- to 2.5-fold increase in the LRRC8A protein content in A2780WT, whereas its expression in A2780CisR was unaffected (44). Recent studies performed by Yang and coworkers (55) have indicated that Cisplatin activates a Cl^−^ current in nasopharyngeal CNE-2Z carcinoma cells, which exhibits properties similar to VRAC (outward rectification, ATP dependence, selectivity sequence of I^−^ > Br^−^ > Cl^−^ > gluconate), and which could be inhibited by the anion channel blocker tamoxifen and extracellular ATP. Studies by Planells-Cases and coworkers (36) indicate that Cisplatin induced taurine release and the cytotoxic effects of the chemotherapeutic drugs Staurosporine, Cisplatin and Carboplatin are abolished in LRRC8A and LRRC8D knockout HEK cells (36). In this context it is noted that osmotic cell shrinkage previously has been shown to induce Caspase-3 activation and apoptosis in EATC via activation of a volume sensory cell death pathway, where p53 is phosphorylated by the protein kinase p38 and hence protected against degradation (11, 27). Consequently, it has been suggested that Cisplatin-induced cell death in A2780WT cells via the intrinsic cell death pathway involves activation of the volume sensory cell death pathway due to activation of transporters for ions/organic osmolytes/AVD (37) and that the reduced Cisplatin sensitivity in A2780CisR partly reflects reduced LRRC8A expression/AVD (44). In congruence the volume sensitive Cl^−^ conductance (VRAC) and the amino acid permeability (VSOAC) have been shown to be reduced and the AVD1 absent in EATC-MDR (37).

In the present work we have used the human ovarian (A2780) and lung (A549) cancer cells and find that LRRC8A contributes to the initial phase of apoptosis induced by genotoxic stress as well as the regulation of p53, MDM2, and p21^Waf1/Cip1^ in Cisplatin-sensitive A2780 and A549 cells. We find that cellular sensitivity toward Cisplatin is reinstalled in the otherwise resistant A2780 cells following expression/reestablishment of LRRC8A activity. LRRC8A downregulation, obtained by a reduction in either total LRRC8A protein expression (A2780) or the fraction expressed in the plasma membrane (A549), was found to be a promoting factor for development of resistance toward Cisplatin-induced apoptosis.

## EXPERIMENTAL PROCEDURES

### 

#### Inorganic solutions.

Phosphate-buffered saline (PBS) contained 137 mM NaCl, 2.6 mM KCl, 6.5 mM Na_2_HPO_4_, and 1.5 mM KH_2_PO_4_. Isotonic (A2780 cells) NaCl solution (300 mOsM) contained 143 mM NaCl, 5 mM KCl, 1 mM Na_2_HPO_4_, 1 mM CaCl_2_, 1 mM MgSO_4_, and 10 mM *N*-2-hydroxyethyl piperazine-*N*′-2-ethanesulfonic acid (HEPES). The 320 mOsM isotonic NaCl solution (for A549 cells) was prepared by increasing the NaCl concentration to 152.5 mM. The hypotonic NaCl solution was prepared by reducing the NaCl concentration to 92.5 mM (200 mOsM) without changing the concentration of the other components. pH was in all solutions adjusted to 7.4. Leu-Lys (yeast) media contained 10% ASD-10, 2% glucose, 0.01% CaCl_2_, 1% leucine and 1% lysine in ddH_2_O (autoclaved before use) and added 0.5% V-200 and 8 ng/ml tetracycline. Lyticase buffer contained 100 mM Tris-HCl, pH 8.0, 100 mM EDTA and 2/3 LB media.

#### Cell culture.

Wild-type (WT) and Cisplatin-resistant (CisR) humane ovarian A2780 and lung A549, as well as human embryonic kidney (HEK-293) cells, were cultured in 75-cm^2^ culture flasks (CellStar, Grenier Bio, Germany) in Roswell Park Memorial Institute (RPMI) 1640 medium or Dulbecco's Modified Eagle medium (DMEM), respectively. All media were supplemented with 10% fetal bovine serum (FBS) and 1% penicillin/streptomycin. The media for A2780 cells were additionally supplemented with 2 mM l-glutamine. Penicillin/streptomycin, RPMI-1640, and DMEM medium, FBS, l-glutamine, and trypsin/EDTA were purchased from Sigma Aldrich (St. Louis, MO). Hypertonic media were obtained by supplementation of the growth media with 150 mM NaCl. All cell cultures were kept at 37°C, 5% CO_2_, and 100% humidity. The cells were subcultured two times a week using 0.25% trypsin/EDTA in PBS. The Cisplatin-resistant A2780 cells (A2780CisR) were, between every third passage, treated with 1 μM Cisplatin to maintain their resistance phenotype. Likewise, resistant A549 cells were continuously treated with either 5 (A549CisR5) or 10 (A549CisR10) μM Cisplatin once a week. Resistant A549 cells (A549CisR5 and A549CisR10) were developed by exposing A549WT cells to an increasing concentration (up to 5 or 10 μM) of Cisplatin for a period of 6 mo. The A2780 cells were a gift from Dr. I. Romero-Canelón (Univ. of Warwick, UK). The HEK-293 lrrc8A^−/−^ (clone E7) was a gift from Prof. Thomas J. Jentsch FMP (Leibniz-Institut fuer Molekulare Pharmakologie) and MDC (Max-Delbrueck-Centrum fuer Molekulare Medizin), Berlin, Germany.

#### Transient knock-down.

Transient knock-down of A2780wt and A549wt cells was carried out using a pool of four siRNA LRRC8A (SMARTpool: ON-TARGETplus, GE Healthcare, Dharmacon), which has previous been used by Voss et al. (49) and siRNA p53 (SignalSilence p53, Cell signaling). MISSION Universal Negative Control siRNA (Sigma Aldrich) was used to create a baseline for knock-down efficiency. Cells grown to 40–50% confluence were transfected with LRRC8A or negative control (scramble) siRNA at a concentration of 25 nM using DharmaFECT-1 Transfection Reagent (Thermo Scientific). For p53 silencing A2780 cells were transfected with 10 nM, 25 nM, or 50 nM siRNA p53 for estimation of the minimal concentration. After 24 h incubation, the medium was replaced by transfection reagent-free medium, and the cells were left for another 24 h. Knockdown efficiency was estimated by Western blot analysis of LRRC8A and by its capability to reduce swelling-induced taurine efflux. In the case of siRNA p53 the cisplatin (10 μM, 24 h) induced increase in the p53 protein expression was found to be reduced to 24%, 31%, and 41% by 10 nM, 25 nM, or 50 nM siRNA p53, respectively. A concentration of 25 nM was chosen for p53 siRNA silencing.

#### LRRC8A-expression vector.

The LRRC8A-GFP expression vector was generated by homologous recombination in yeast. Yeast cells were transformed with BamHI. SalI and HindIII (Fermentas, Thermo Fischer Scientific) digested pPAP7160 vector (14) and a LRRC8A DNA fragment. The LRRC8A cDNA fragment was obtained by PCR using AccuPol polymerase (VWR, Denmark), a human LRRC8A cDNA ORF clone (RC208632, OriGene) and the following primers: LRRC8A (forward) 5′-ATA TAA GCA GAG CTG GTT TAG TGA ACC GTC AGA TCG GGT TGA ACC ATG ATT CCG GTG ACA GAG CT-3′and LRRC8A (reverse) 5′-ACC CCG GTG AAC AGC TCC TCG CCC TTG CTC ACC ATG GCC TGC TCC TTG TCA GC-3′ (Tag Copenhagen A/S, Denmark). The 5′ LRRC8A PCR product carried a 35-nucleotide sequence identical to the pPAP7160 vector upstream to the restriction enzyme digestion-site while the 3′ LRRC8A PCR product contained a 35-nucleotide sequence identical to the 5′ coding sequence of EGFP on the pPAP7160 vector. Following transformation the yeast cells were lysed by centrifugation (3.000 rpm, 5 min), addition of 1:1 lyticase buffer and 10 μl lyticase (5 unit/μl in TE buffer, Sigma). Cells were incubated 1 h at 37°C with thorough vortexing. After incubation, the cells were added to 10 μl 20% SDS, mixed, and frozen (−20°C). The LRRC8A-expression vector was purified using a NucleoSpin Plasmid DNA purification kit (Macherey-Nagel, Germany). DNA yield was amplified by transformation into *E.coli* (OmniMax cells), followed by NucleoBond Xtra Maxi Plasmid DNA Purification (Macherey-Nagel, Germany). The correct nucleotide sequence of the LRRC8A vector was confirmed by DNA sequencing. The functionality of the LRRC8A-GFP expression vector was verified in human embryonic kidney LRRC8A KO cells (LRRC8A^−/−^, kindly provided by Prof. Thomas J. Jentsch) where it was determined that LRRC8A expression (verified by Western blot, *n* = 4, see technique below) and maximal swelling-induced taurine release (verified by tracer technique, *n* = 3, see technique below) were increased 17.5 ± 7.4-fold and 7.9 ± 2.1-fold, respectively, following transfection with 0.05 ng/μl LRRC8A-GFP vector and 1.2 ± 0.4-fold and 1.9 ± 0.4-fold, respectively. following transfection with 0.05 ng/μl empty-GFP vector.

#### SDS-PAGE and Western blotting.

SDS-PAGE and Western blotting were used to quantify changes in protein levels of LRRC8A (94 kDa), p53 (53 kDa), Bax (20 kDa), p21^Waf1/Cip1^ (p21^CDKN1A^, 21 kDa), Noxa (10 kDa), MDM2 (90 kDa), phosphor-MDM2 (Ser166), ATM (350 kDa), phospho-ATM (Ser1981), p42/p44 (Erk1/2, 42/44 kDa), phospho-p44/42 MAPK (Erk1/2) (Thr202/Tyr204), human Caspase-9 (35, 37, and 47 kDa), and the housekeeping protein β-actin (42 kDa), histone H3 (17 kDa), or α-tubulin (52 kDa). Protein extraction and blotting were performed on cells grown to 80–90% confluence in 6-cm Petri dishes or 6-well culture plates. Cells were gently washed once in ice-cold PBS and subsequently lysed in lysis buffer containing 1% SDS, 10% glycerol, 150 mM NaCl, 20 mM HEPES, 1 mM EDTA, 0,5% Triton X-100, 1 mM Na_3_VO_4_ and 1% protease inhibitor cocktail. Lysates were briefly sonicated and subsequently centrifuged for 5 min at 5°C and 20,000 rpm to separate the proteins extracts from insoluble cell material. The protein content was estimated using a Bio-Rad DC protein assay (Bio-Rad, Hercules, CA). Lysates were diluted in ddH_2_O (20–40 μg per loading), mixed with NuPAGE sample buffer including dithiothreitol (DTT), and proceeded to SDS-PAGE gel electrophoresis (NuPAGE precast 10% or 4–12% Bis-tris gels in NuPAGE MOPS SDS running buffer, Invitrogen, Waltham, MA) in NOVEX chambers under reducing and denaturing conditions. A benchmark protein ladder (Invitrogen) was used to indicate the molecular weight. Following electrophoresis, NuPAGE transfer buffer (Invitrogen) was used for protein transfer to nitrocellulose membranes. Proper protein transfer was verified by Ponceau-S staining. Unspecific membrane-binding were blocked by incubation in TBST (0.01 M Tris-HCl, 0.15 M NaCl, 0.1% Tween 20, pH 7.4) containing 5% nonfat dry milk at 37°C for 1 h on a shaking table. Membranes were incubated with primary antibodies diluted in blocking buffer overnight at 4°C. Next, the membranes were washed in TBST and subsequently incubated with secondary antibodies for 1 h at room temperature. The monoclonal mouse anti-human-LRRC8A (SAB1412855), anti-human-p21^Waf1/Cip1^ (P1484), and anti-β-actin (A1978) antibodies were used in a dilution of 1:250 (LRRC8A and p21) and 1:1,000 (β-actin) and purchased from Sigma-Aldrich. Noxa (no. 14766), ATM (no. 2873), phospho-ATM (no. 13050), phosphor-MDM2 (no. 3521), Bax (no. 2772), p53 (no. 2524), Caspase-9 (no. 9502), Histone H3 (no. 9717), α-Tubulin (no. 2125), and phospho-p53 (no. 9284) antibodies were from Cell Signaling (Danvers, MA) and used in a dilution of 1:250 (Noxa, phosphor-MDM2, Bax, Caspase-9, Histone H3, α-tubulin and phosphor-p53) or 1:100 (ATM, phosphor-ATM and p53), respectively. The antibody against MDM2 (sc-965) was from Santa Cruz Biotechnology and used in the dilution of 1:100. The antibody against the COOH-terminal part of the Na^+^/K^+^-ATPase antibody was made and kindly donated by Prof. Per Amstrup-Pedersen (Univ. of Copenhagen, Denmark) and used in the dilution 1:250. The secondary AP-conjugated anti-mouse and anti-rabbit antibodies (Sigma) were both used in a dilution of 1:5,000. Following final washes in TBST, membranes were developed using BCIP/NBT (KPL, Gaithersburg, MD), scanned and bands quantified using UN-SCAN-IT (Silk Scientific).

#### Cell surface biotinylation and membrane protein isolation.

A549WT and CisR10 cells, grown to 80% confluence in four T75 flasks (each), were biotinylated, lysed and labeled proteins isolated using Pierce Cell Surface Protein Isolation Kit (Thermo Fisher Scientific) following manufacturer's instructions. SDS-PAGE and Western blotting were used to analyze the amount of LRRC8A, Na^+^/K^+^-ATPase (positive plasma membrane control/loading control), and Histone 3 (nuclear control) in both whole cell lysate and purified samples.

#### Cell viability assay-MTT.

Conversion of the yellow soluble tetrazolium salt 3-(4,5-dimethylthiazol-2-yl)-2,5-diphenyltetrazolium bromide (MTT from Sigma) into a blue insoluble formazan was used to determine cell viability after treatment of WT and resistant cells with 10 μM Cisplatin for 48 h. Cells were seeded to a density of 10,000 (A2780) or 5,000 (A549) cells/200 μl cell culture media per well in 96-well plates and incubated for 96 h (37°C, 5% CO_2_, 100% humidity). Cells were after 48 h transiently transfected with 25 nM siRNA (human LRRC8A siRNA or 2 ng/μl scramble siRNA) or a vector (either expressing LRRC8A-GFP or an empty vector only carrying the GFP gene) using DharmaFECT-1 Transfection Reagent (Thermo Scientific). After 24 h incubation, media were changed to normal growth medium or medium containing 10 μM Cisplatin and/or 400 μM DIDS. At the end of the incubation period, 100 μl was removed, and 25 μl of a MTT solution (5 mg/ml MTT in sterilized PBS) was added to each well, and the plate was incubated in the cell culture incubator for 3 h. Following incubation 100 μl fresh-made 10 mM HCl containing 1% SDS was added to each well and incubated overnight in a fume hood to solubilize the colored formazan crystals. Samples were measured at 570 nm using Wallac Envision Multilabel plate reader (PerkinElmer). Data were represented relative to the absorbance from the respective untreated control cells. Each experiment was performed in triplicate.

#### Caspase-3 colorimetric protease assay.

Wild-type, resistant, and transiently transfected A2780 cells were grown to 80% confluency in T25 culture flasks and incubated in the presence or absence of 10 μM NS3728 or 400 μM DIDS in combination with 10 μM Cisplatin, 20 ng/ml TNFα, or 150 mM NaCl (600 mOsM) for 18 or 4.5 h. Cells were carefully washed once in ice-cold PBS, trypsinized, and transferred to 15 ml falcon tubes with RPMI-1640 (A2780). Next, the cell suspensions were centrifuged at 1,000 *g* for 5 min at room temperature, and the extracellular medium was carefully removed to collect all cellular material including apoptotic bodies. The cell suspensions were then washed one additional time in PBS, centrifuged as before and cell pellet carefully collected. The cells were lysed in ice-cold lysis buffer for 15–20 min, transferred to Eppendorf microcentrifuge tubes, and centrifuged (5 min at 10,000 *g*, 4°C) to precipitate nonsoluble cell material. The supernatants were transferred to new Eppendorf tubes and protein concentrations were estimated using a Bio-Rad DC protein assay with bovine serum albumin (BSA) as a standard. Lysates, which were not used immediately, were stored at −80°C until use. The protein content was, before activity measurements, adjusted to 4 μg/μl. Caspase-3 activity in cell lysates was estimated in black 96-well plates with transparent bottom using the ApoTarget Caspase-3/CPP32 Colorimetric assay (Invitrogen, Taastrup, Denmark) according to the manufacturer's manual by measuring protease activity toward the peptide substrate acetyl-Asp-Glu-Val-Asp p-nitroanilide (Ac-DEVD-pNA) and estimating the p-nitroanilide (pNA) production. Absorbance was measured at 405 nm using a Wallac Envision Multilabel plate reader (Perkin Elmer). Experiments were performed in duplicate.

#### Caspase-9 activity.

The intrinsic proteolytic Caspase-9 activity was determined by SDS-PAGE and Western Blotting (see above).

#### Estimation of taurine efflux.

Swelling-induced taurine efflux was estimated at room temperature on cells grown to 80% confluence in 6 well polyethylene culture plates as previously described (26). Cells were loaded with [^3^H]taurine (PerkinElmer, 37,000 Bq/well) in complete growth medium for 2 h (37°C, 5% CO_2_, 100% humidity). Before the experiment, the growth media was removed and each well (each representing one experiment) was washed 3 times with 1 ml isotonic NaCl medium (300 or 320 mOsm) to remove residual extracellular isotope and growth media. The experiment was performed at room temperature by transferring the NaCl medium from each well to individual scintillation vials (Snaptwist Scintillation vial, 6.5 ml, VWR) and replacing it with fresh medium at 2-min intervals. The experiment was run for the total of 30 min, in the absence or presence of inhibitors, i.e., the specific VRAC/VSOAC blocker NS3728 (a gift from NeuroSearch, Denmark) and DIDS (4,4′-diisothiocyanatostilbene-2,2′-disulfonate, dissolved in ddH_2_O, Sigma Aldrich). At time 12 min, isotonic medium was replaced by hypotonic medium (200 mOsm). At the end of the experiment, remaining intracellular isotope was extracted by addition of 1 ml 1 M NaOH (1 h, shaking table) followed by two additional washes with ddH_2_O. Each vial was added to 3.5 ml scintillation fluid (Parkard Ultima Gold, PerkinElmer), mixed carefully, and proceeded to β-scintillation counting in a Perkin Elmer scintillation counter (Waltham, MA). The total ^3^H activity in the cell system was calculated as the sum of ^3^H activity released during the efflux experiment and in the NaOH/water washouts. The fractional taurine release rate constant (*k*, min^−1^) was calculated from the equation: *k* = [ln(*X*_1_) − ln(*X*_2_)]/(*t*_1_ − *t*_2_), where *X*_1_ and *X*_2_ denote the fraction remaining in the cell at time *t*_1_ and *t*_2_, respectively.

#### Statistics.

All data were statistically tested (SigmaPlot version 12) by one-way ANOVA with Fisher LSD Method as post test and/or by Student's *t*-test. In bar- and scatterplots the error bars signify standard error of the mean (SE).

## RESULTS

**Fig. 1.**
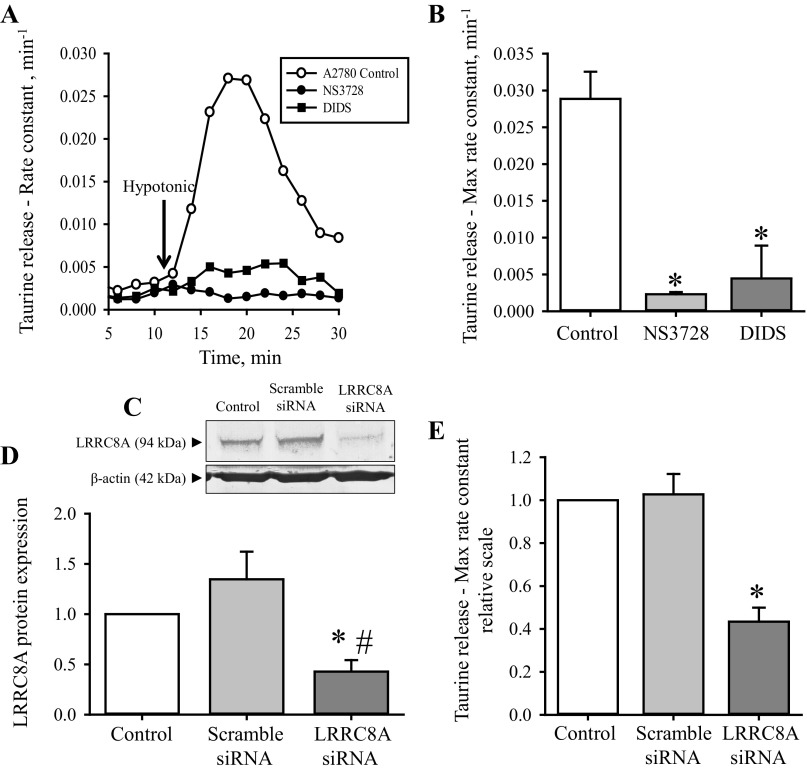
Impairment of swelling induced taurine release following pharmacological inhibition or transient knock-down of LRRC8A in wild-type A2780 cells. Volume-sensitive taurine release and LRRC8A protein expression were measured in ovarian A2780WT cells by tracer technique and Western blot analysis, respectively. A2780WT cells were loaded with [^3^H]taurine for 2 h, washed, and exposed to isosmotic NaCl medium (300 mOsM) for 10 min and subsequently to hyposmotic medium (200 mOsM) for 20 min (arrow in *A* indicates shift to hypotonicity). Samples were taken every second minute. *A*: fractional rate constant (min^−1^) for taurine release was determined and plotted vs. time (min) under isosmotic and hyposmotic conditions in the absence (○) or presence of the VRAC/VSOAC blockers NS3728 (●) or DIDS (■). The anion blockers NS3728 and DIDS were used in the concentration of 10 μM (≈2.5 μM free) and 100 μM, respectively, and present during the isotonic/hypotonic release experiment. Data represent 1 of 3 sets of experiments. *B*: maximal rate constant for taurine release was determined 6 min after hyposmotic exposure as illustrated in *A*. Data represent the mean maximal rate constants determined from 3 sets of experiments ± SE. *Significantly reduced compared with control (Student's *t*-test). *C*: efficiency of siRNA-mediated LRRC8A knock-down in A2780WT cells was determined by Western blot analysis using control, scramble siRNA and LRRC8A siRNA transfected cells and specific monoclonal antibody raised against human LRRC8A and β-actin (loading control). *D*: LRRC8A/β-actin protein band intensity ratios were calculated from Western blots shown in *C* and values given relative to control cells. Data represent mean of 4 experiments ± SE. * and #: Significantly reduced compared with Control and Scramble siRNA, respectively (Student's *t*-test). Scramble siRNA was not significantly larger than Control (*P* = 0.15, Student's *t*-test). *E*: maximal rate constant for taurine release was determined at time 6 min after hypotonic exposure in A2780WT (open bar) cells or in A2780 cells exposed to scramble siRNA (light gray bar) or siRNA directed against human LRRC8A (dark gray bar). Data represent mean of 4 experiments ± SE. *Significantly reduced compared with Control (Student's *t*-test).

Several groups have recently shown that LRRC8A significantly contributes to VRAC/VSOAC channel activity either by being a regulatory subunit or a critical channel pore-forming component (36, 39, 49). With respect to drug resistance, data from our group have indicated that development of resistance in human ovarian A2780 carcinoma cells correlates with a reduced LRRC8A protein expression, and an inability to activate VSOAC and to volume-regulate in response to hyposmotic cell swelling (44). Here we establish an in vitro cell-based assay by which it is possible to investigate the direct involvement of LRRC8A-mediated channel activity in development of resistance against Cisplatin. For this purpose, we use volume-sensitive release of the organic osmolyte taurine to track VSOAC activity (28), pharmacological inhibitors, which are known to inactivate VRAC/VSOAC, and commercially available siRNAs, which have previously been used to selectively knock-down human LRRC8A (49). From Fig. 1*A*, it is seen that acute exposure to the two anion-channel inhibitors NS3728 and DIDS abolishes the swelling-induced taurine efflux in ovarian A2780WT cells. Both NS3728 and DIDS were found to reduce the maximal taurine rate constant more than 90% compared with the untreated control cells (Fig. 1*B*). These data are in accordance with what has previously been shown for this and other cell lines (19, 44). As NS3728 and DIDS are also known to affect the activity of, e.g., anion channels like the Ca^2+^-dependent Anoctamin channels (15, 23, 42), we used siRNA directed against human LRRC8A to specifically knock-down VRAC/VSOAC channel activity. Figure 1, *C* and *D*, shows a representative Western blot from a knock-down experiment and the corresponding quantification, respectively. From these figures it is seen that we successfully managed to knock-down the LRRC8A protein content to 40% in A2780WT cells. To test the effect of LRRC8A knock-down in A2780 cells, we determined the ability of the knock-down cells to release taurine in response to hyposmolality (Fig. 1*E*). These data likewise confirm the previous assumption that LRRC8A significantly contributes to VRAC/VSOAC channel activity and that the observed knock-down efficiency seen by Western blot analysis (Fig. 1, *C* and *D*) correlates with the reduction in swelling-induced taurine efflux (Fig. 1*E*).

### 

#### Eighteen-hour exposure to anion- and cation-channel blockers increases LRRC8A protein content in wild-type A2780 cells.

**Fig. 2.**
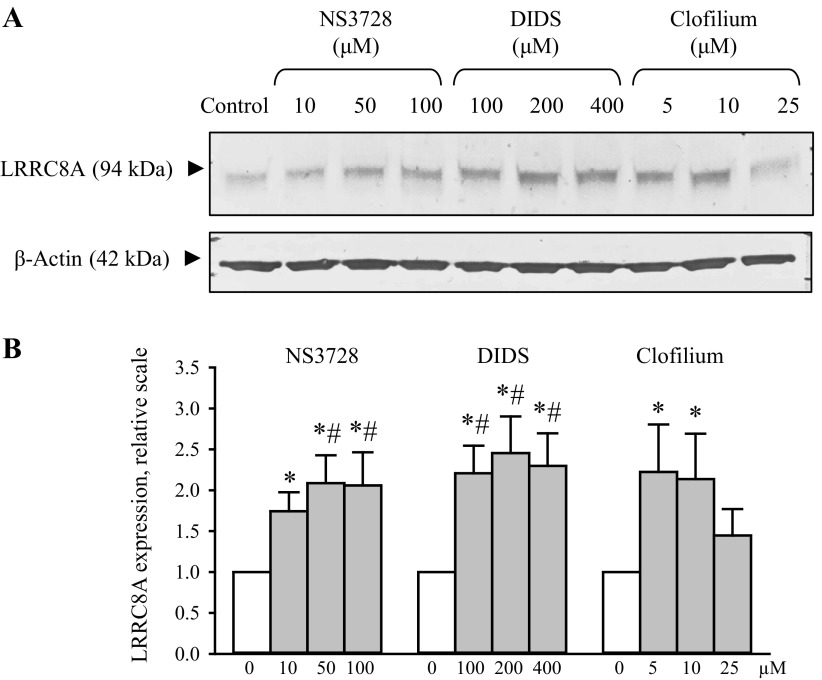
Eighteen-hour exposure to anion- and cation-channel blockers leads to increased LRRC8A protein expression in wild-type A2780 cells. Wild-type A2780 cells were exposed to increasing concentrations of the anion- and cation-channel blockers (NS3782, DIDS, and Clofilium) for 18 h. *A*: lysates were taken and used for Western blot analysis using a monoclonal antibody raised against human LRRC8A and β-actin (loading control). *B*: LRRC8A/β-actin protein band intensity ratios were calculated from Western blots shown in *A* and values given relative to control cells. Data represent mean of 7 sets of experiments ± SE. # and *: Significantly increased compared with Control without inhibitor when tested by ANOVA, Fisher LSD method and Student's *t*-test, respectively.

We have tested the effect of increasing pharmacological channel blockage on LRRC8A protein expression. Figure 2 shows that exposing A2780WT cells for 18 h to an increasing concentration of the anion channel blockers NS3728 and DIDS results in an increased LRRC8A protein expression, indicating that the A2780 cells apparently compensate for an acute reduction in LRRC8A channel activity by upregulating the protein-expression of the channel proteins. We also find that inhibition of K^+^ channels with clofilium increases LRRC8A expression in A2780 cells (Fig. 2). Whether the latter effect is related to a concomitant depolarization of the membrane potential and hence a decrease in the driving force for transport of anions through the LRRC8A channel complex was not investigated further.

#### Administration of anion- and cation-channel blockers abolishes Cisplatin-induced increase in p53/p21^Waf1/Cip1^ protein expression in wild-type A2780 cells.

**Fig. 3.**
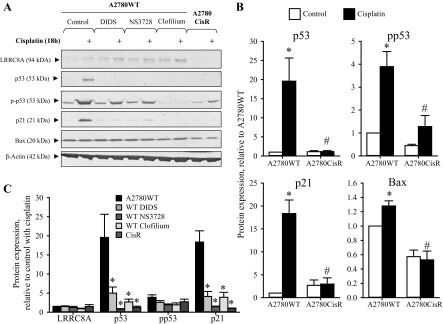
Administration of anion- and cation-channel blockers reduces Cisplatin-induced expression of p53 and p21^Waf1/Cip1^ in wild-type A2780 cells (A2780WT) to the same level as observed in Cisplatin-resistant A2780 cells (A2780CisR). Whole cell protein lysates for Western blot analysis were obtained from A2780WT and A2780CisR cells exposed to 10 μM Cisplatin for 18 h in combination with the anion/amino acid channel blockers NS3728 (100 μM)/DIDS (400 μM) or the cation-channel (TASK-2) blocker clofilium (25 μM). Only A2780WT cells were exposed to channel blockers. Protein expression of LRRC8A, p53, p-p53 (Ser15), p21, and Bax was determined using specific antibodies (see experimental procedures). β-Actin was used as loading control. *A*: representative Western blot. *B*: p53, pp53, p21, and Bax protein expression relative to β-actin in A2780WT and A2780CisR (open bars) control cells and following Cisplatin exposure (black bars). Data represent 4–9 individual sets of experiments where ratios are given relative to the untreated WT cells ± SE. * and #: Expression is significantly increased in Cisplatin-treated cell compared with control cells and significantly reduced in Cisplatin-treated A2780CisR cells compared with Cisplatin-treated A2780WT cells, respectively (ANOVA, Fisher LSD method). *C*: graph represents the relative effect of Cisplatin (Cisplatin-treated/respective untreated control) on LRRC8A, p53, p-p53, and p21 protein expression in A2780WT, A2780CisR (CisR), and A2780WT cells cotreated with the anion- and cation-channel blockers. Data represent mean of 4–9 individual experiments ± SE. *Significantly reduced effect of Cisplatin compared with A2780WT (ANOVA, Fisher LSD method).

It has previously been shown that acute hyperosmotic stress leads to a biphasic stabilization and activation of the transcription factor p53 in mouse NIH3T3 fibroblasts, supposedly through Ser-15 phosphorylation of p53 by the serine/threonine kinase ATM and/or the p38 MAP kinase (11, 27). As exposure to Cisplatin likewise elicits cell shrinkage under isotonic conditions, we tested the effect of Cisplatin on p53-mediated signaling. From Figs. 3*A* (Western blots) and Fig. 3*B* (protein quantification), it is seen that 18 h exposure to Cisplatin, similar to osmotic cell shrinkage, leads to an increased p53 protein expression/phosphorylation (Ser15) as well as an enhancement of the expression of downstream elements to p53, e.g., p21^Waf1/Cip1^ and a minor increase in Bax in A2780WT cells. From Fig. 3, *A* and *B*, it is also seen that the Cisplatin-induced p53 protein expression and downstream signaling is almost absent in the corresponding Cisplatin-resistant subtype A2780CisR, although a minor, significant increase in p53 phosphorylation is observed. As the protein expression of LRRC8A has previously been found markedly downregulated in A2780CisR (44), we wanted to test whether administration of the pharmacological VRAC/VSOAC blockers NS3728 and DIDS might give rise to the same phenotype in A2780WT cells, as observed in A2780CisR. From Fig. 3*C* it is seen that Cisplatin cotreatment with either NS3728 or DIDS significantly reduces p53 and p21^Waf1/Cip1^ protein level in A2780WT cells, i.e., the wild-type A2780 cells resemble A2780CisR in the presence of the inhibitors. Ser-15 phosphorylation of p53 was affected as well in A2780WT by the inhibitor although not to the same extent as observed for total p53 and p21^Waf1/Cip1^. This could indicate that VRAC/VSOAC activity is not important for the phosphorylation of p53, but rather regulates its expression and/or stability independently of the phosphorylation. As the activity of VRAC/VSOAC is highly dependent on the coactivity of potassium channels like TASK-2 to maintain a stable membrane potential, we wondered if the TASK-2 channel blocker clofilium might also affect Cisplatin-induced p53 stabilization/activation and downstream signaling. Supportively, we found that clofilium also prevents the increase in p53 protein expression and hinders its induction of p21^Waf1/Cip1^ (Fig. 3*C*). These data indicate that coactivity of anion- and cation-channels, which leads to cell shrinkage, is necessary for Cisplatin-induced stabilization/activation of p53 and its downstream signaling involving increased expression of p21^Waf1/Cip1^.

**Fig. 4.**
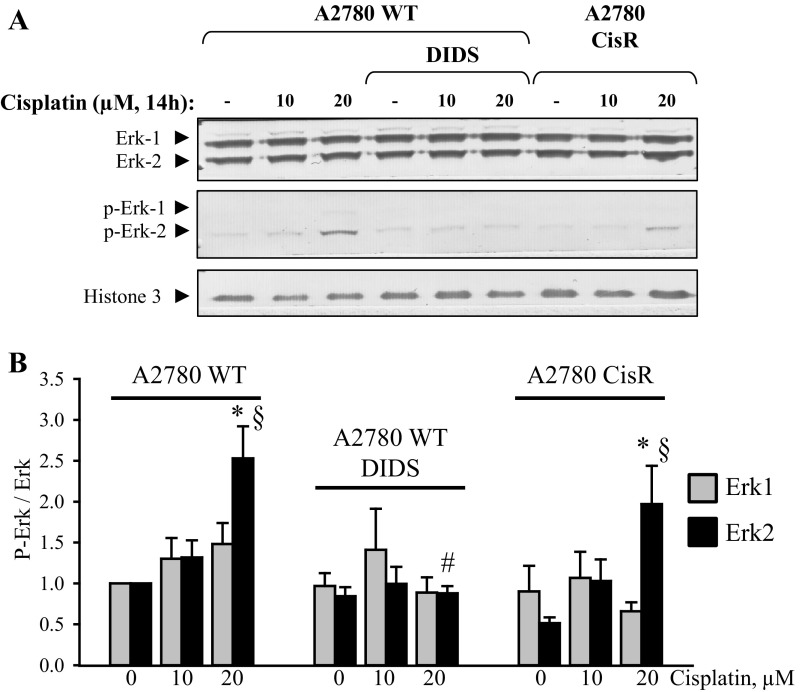
The anion-channel blocker DIDS reduces Cisplatin-induced Erk-2 phosphorylation in A2780 cells. Whole cell protein lysates for Western blot analysis were obtained from A2780WT and A2780CisR cells exposed to 0, 10, or 20 μM Cisplatin for 14 h in combination with the anion/amino acid channel blockers DIDS (400 μM). Only A2780WT cells were exposed to DIDS. Total Erk-1/-2 protein and phospho-Erk-1/-2 expression were determined using specific antibodies (see experimental procedures). Histone-3 (H3) was used as loading control. *A*: representative Western blot. *B*: phospho-Erk-1 protein expression relative to total-Erk-1 (gray bars) and phospho-Erk-2 protein expression relative to total-Erk-2 (black bars) in A2780WT, A2780WT cotreated with DIDS, and A2780CisR control cells and following 14 h Cisplatin exposure. Data represent 5 individual sets of experiments, where ratios are given relative to the untreated WT cells ± SE. * and § indicate that the Erk-phosphorylation ratio is significantly increased in Cisplatin-treated cells when compared with the respective control cells; # indicates that the Erk-phosphorylation ratio in cisplatin-treated A2780WT cells is significantly reduced by DIDS (ANOVA, Fisher LSD method).

Studies by Wang and colleagues (52) indicate that Cisplatin-induced apoptosis requires activation of the MAP kinase signaling pathway in a process involving growth receptor transactivation, and downstream activation of MEK1/2 and Erk1/3 in HeLa and A549 cells. Woessman and coworkers (53) find that Ras-mediated activation of ERK by cisplatin induces cell death independently of p53 in osteosarcoma and neuroblastoma cell lines. In contrast, Persons and coworkers (34) find that p53 coimmunoprecipitates with ERK1/2 and that inhibition of ERK1/2 activation reduces Cisplatin-mediated p53-phoshorylation (Ser^15^), i.e., Cisplatin induced ERK1/2 signaling is an upstream event to p53 activation in A2780 cells. Figure 4 shows that exposure to 20 μM Cisplatin for 14 h indeed leads to an increased phosphorylation of Erk2 in both A2780WT and A2780CisR. Total Erk1 and Erk2 protein expression is unaffected by Cisplatin. Cotreatment with the anion channel blocker DIDS completely abolished the Erk2 phosphorylation in A2780WT, indicating that VSOAC/VRAC might also be a regulator of the MAP kinase signaling pathway. We find that only treatment with Cisplatin concentrations above 20 μM Cisplatin, in correspondence with Wang and coworkers findings, leads to an increased phosphorylation of Erk1/2. This indicates that Cisplatin-induced activation of Erk1/2 may be less important, as the plasma Cisplatin concentration in cancer patients, treated with 100 mg Cisplatin/m^2^, stabilizes at 10 μM (16). The involvement of Erk1/2 was therefore not investigated further.

#### Cotreatment with anion-channel blockers and transient knock-down of LRRC8A eliminate Cisplatin-induced, but not TNFα and hyperosmotic-induced Caspase-3 activation in wild-type A2780 cells.

**Fig. 5.**
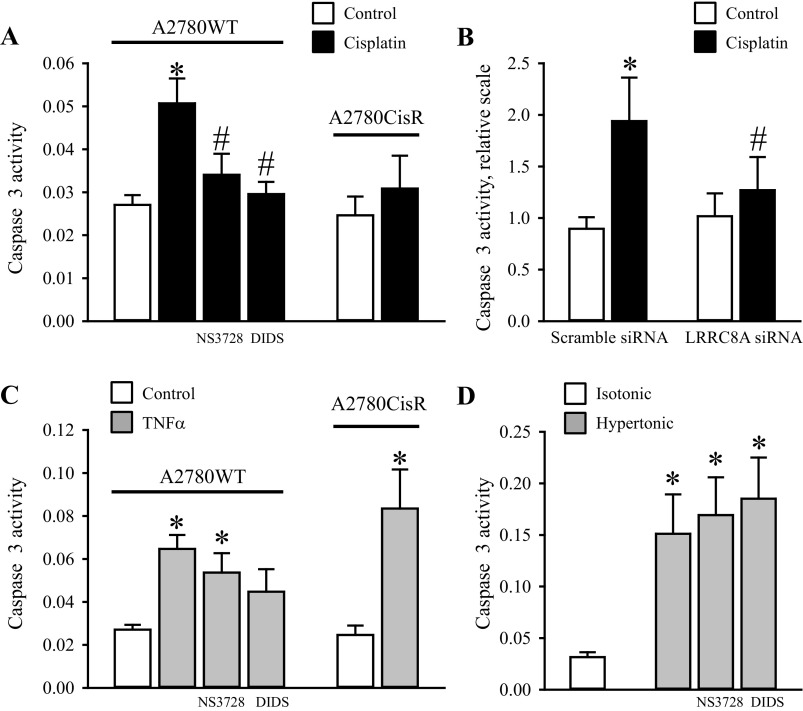
Cotreatment with anion-channel blockers and transient knock-down of LRRC8A abolish Cisplatin-induced but not TNFα and Hyperosmotic-induced Caspase-3 activation in wild-type A2780 cells. A2780WT and A2780CisR cell lysates were used for Caspase-3 activity assay using a commercial kit (see experimental procedures). *A*: A2780CisR cells were exposed to 10 μM Cisplatin for 18 h and A2780WT cells were likewise treated with Cisplatin in the absence or presence of 100 μM NS3728 or 400 μM DIDS. Data represent 6–8 (A2780WT) and 4 (A2780CisR) individual sets of experiments ± SE. * and #: Significantly increased by Cisplatin compared with the respective control and significantly reduced compared with Cisplatin treatment in the absence of the inhibitor, respectively (ANOVA, Fisher LSD method). *B*: A2780WT were treated with either 25 nM scramble siRNA or LRRC8A siRNA for 30 h and subsequently exposed to 10 μM Cisplatin for another 18 h. Data represent 4 individual experiments ± SE. * and #: Significantly increased by Cisplatin compared with the respective control and significantly reduced compared with Cisplatin treatment in Scramble siRNA-treated cells (Student's *t*-test). C: A2780CisR cells were exposed to 20 nM TNFα for 18 h. A2780WT cells were likewise treated with TNFα in the absence or presence of 100 μM NS3728 or 400 μM DIDS. Data represent 6–8 (WT) and 4 (CisR) individual experiments ± SE. *Significantly increased compared with the respective control (ANOVA, Fisher LSD method). *D*: A2780WT cells were exposed to isotonic or hypertonic NaCl for 4.5 h in the absence or presence of 100 μM NS3728 or 400 μM DIDS. Data represent 3 individual experiments ± SE. *Significantly increased compared with isotonic control (ANOVA, Fisher LSD method).

An increase in Caspase-3 activity has previously been used as an indicator of apoptotic progress, and it is commonly known that p53 activity is an upstream event for cleavage and activation of Caspase-3 in response to several types of cellular stress, e.g., Cisplatin-induced genome toxicity and hyperosmolarity (11, 27). From Fig. 5*A* it is seen that 10 μM Cisplatin (18 h) induces a 2-fold increase in Caspase-3 activity in A2780WT, whereas Caspase-3 activity in A2780CisR is unaffected by Cisplatin. Furthermore, we find that VRAC/VSOAC inhibition with NS3728/DIDS (Fig. 5*A*) reduces the Cisplatin-induced Caspase-3 activation in A2780WT to the level we observed in A2780CisR. Transient knock-down of LRRC8A (Fig. 5*B*) abolishes the Cisplatin-induced Caspase-3 activation (Fig. 5*B*). These data illustrate that downregulation of LRRC8A in A2780WT results in resistance against Cisplatin-induced apoptosis. To identify at which signaling-level VRAC/VSOAC is involved in the apoptotic pathway, we investigated the involvement of VRAC/VSOAC channel activity on the apoptotic pathway initiated by other types of apoptotic stimulation, e.g., by exposure to TNFα (extrinsic pathway) and hyperosmolality (volume-sensory pathway). From Fig. 5*C* it is seen that TNFα induces a 3- to 3.5-fold and 4.5-fold increase in Caspase-3 activity in A2780WT and A2780CisR cells, respectively. These data thereby confirm our previous results that A2780CisR still enters apoptosis following TNFα exposure (44). When testing the TNFα effect during anion-channel blockage, we found only a slight tendency for NS3728 and DIDS to reduce TNFα-induced apoptosis, which is taken to indicate that VRAC/VSOAC activity is not necessary for death-receptor stimulated Caspase-3 activation in A2780 cells. With respect to Caspase-3 activation induced by hyperosmolality, we found that cell shrinkage per se leads to a 6-fold increase in Caspase-3 activity, and that neither NS3728 nor DIDS had any effect on the caspase stimulation (Fig. 5*D*). Hence, hyperosmotic Caspase-3 activation happens independently of VRAC/VSOAC activities and most probably directly as a consequence of the shrinkage of the cells.

#### Pharmacological inhibition of VSOAC activity protects A2780WT cells against Cisplatin-induced reduction in cell viability, whereas an artificial expression of LRRC8A in A2780CisR restores Cisplatin-sensitivity.

**Fig. 6.**
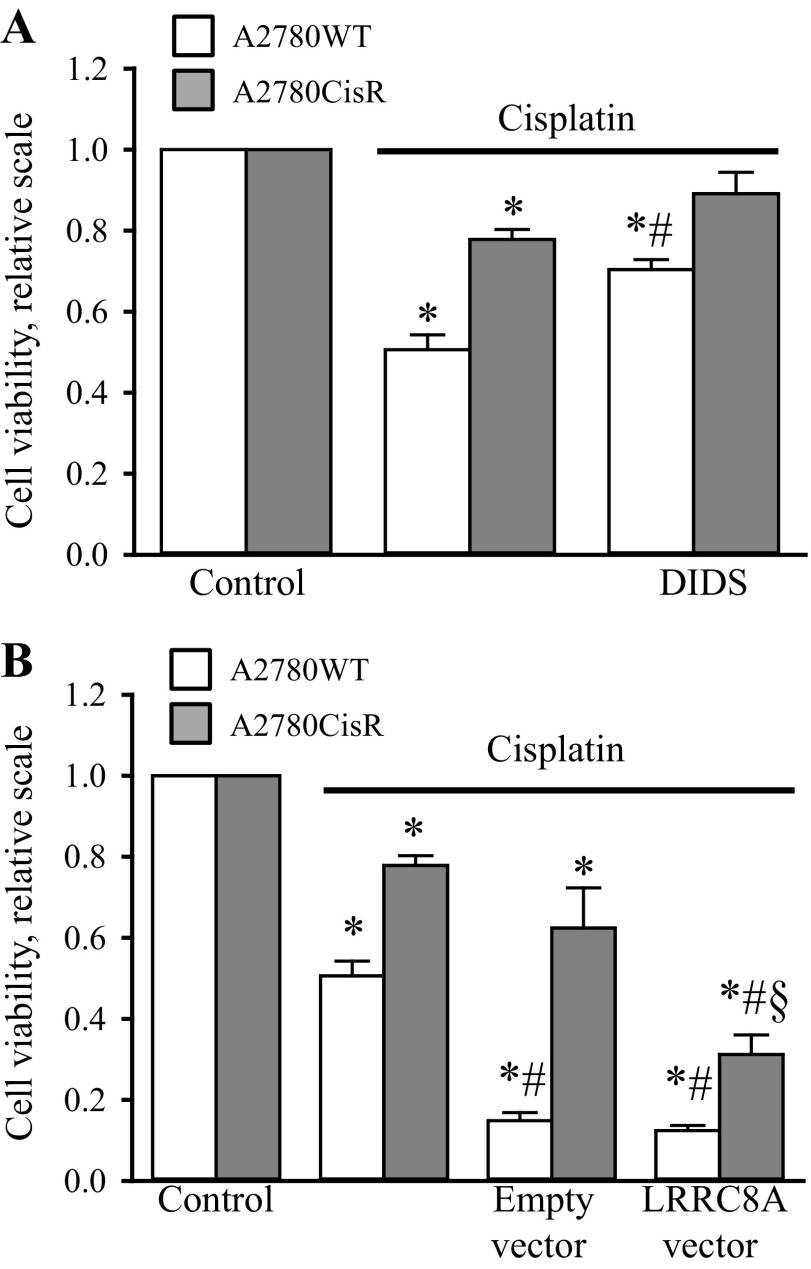
Cell viability of A2780 after Cisplatin treatment is determined by LRRC8A. The cell viability in A2780WT and A2780CisR was measured by MTT (see experimental procedures). A: A2780CisR cells were exposed to 10 μM Cisplatin for 48 h and A2780WT cells were likewise treated with Cisplatin in the absence or presence of 400 μM DIDS. Data represent 5 (A2780WT) and 3 (A2780CisR) individual sets of experiments ± SE. * and #: Significantly decreased by Cisplatin compared with the respective control and significantly increased compared with Cisplatin treatment in the absence of the inhibitor, respectively (Student's *t*-test). *B*: A2780WT and A2780CisR were transfected with either an empty vector or a vector carrying the LRRC8A gene-information. Following 1 day transfection, half of the cells were treated with 10 μM Cisplatin and left for another 48 h before measuring the viability. Data represent 6 (A2780WT) and 4 (A2780CisR) individual sets of experiments ± SE. * and #: Significantly decreased by Cisplatin and vector expression compared with the respective control, respectively (Student's *t*-test). §Significantly reduced by LRRC8A expression compared with empty vector expression (Student's *t*-test).

To verify the cellular effect of LRRC8A and pharmacological blockage of VSOAC on Cisplatin cytotoxicity, we performed a MTT cell viability assay. From Fig. 6, *A* and *B*, it is seen that treatment of A2780 cells with 10 μM Cisplatin for 48 h reduces cell viability to ∼50% in A2780WT cells, whereas an equivalent treatment of A2780CisR only reduces cell viability to ∼80%. Cotreatment with DIDS increases cell viability after Cisplatin treatment in both A2780WT and A2780CisR (Fig. 6*A*), confirming that the cytotoxic effects of Cisplatin are highly dependent on VSOAC activity. As A2780CisR was previously shown to have a significantly reduced LRRC8A protein expression compared with A2780WT (44), we tested whether Cisplatin cytotoxicity could be restored in A2780CisR by artificially increasing the expression of LRRC8A. Figure 6*B* shows that expression of a vector carrying a constitutively active promoter for a LRRC8A gene reduces cell viability in A2780CisR following Cisplatin treatment. Hence, LRRC8A reinstallation renders A2780CisR sensitive to Cisplatin. It is noted that expression of the LRRC8A vector has no effect on cell viability in A2780WT cells as LRRC8A activity is ensured by the endogenous LRRC8A expression. The low viability in cells treated with Cisplatin plus vector most probably reflects the transfection procedure.

#### Abrogation of prolonged cisplatin treatment restores Cisplatin-sensitivity in A2780CisR within 3–6 wk.

**Fig. 7.**
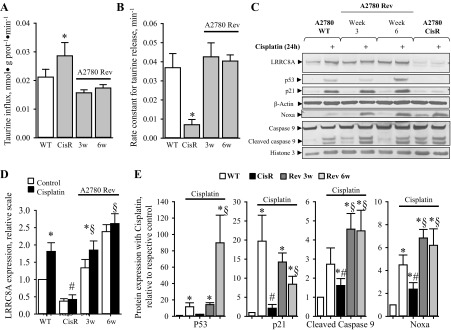
The Cisplatin induced phenotype of A2780CisR is reversible. Continued Cisplatin treatment of A2780CisR was stopped by omission of Cisplatin from the growth medium, and cellular characteristics (taurine influx, swelling induced taurine efflux, and response to Cisplatin treatment) in the progressing cell line, designated A2780Rev, were determined 3 and 6 wk (3w/6w) after redraw of Cisplatin. *A*: cellular taurine influx (nmol·g protein^−1^·min^−1^) was determined under isotonic conditions by tracer technique in A2780WT, resistant cells (CisR), and reversing (A2780 Rev) cells. Taurine influx (nmol·g protein^−1^·min^−1^) was determined from the slope of time traces. Values for A2780CisR and A2780Rev cells (gray bars) are given relative to A2780WT cells (open bars) and represent means of 3 sets of experiments. *Significantly increased compared with WT cells. *B*: fractional rate constant (min^−1^) for taurine release was determined and plotted vs. time (min) under isosmotic and hyposmotic conditions. The maximal rate constant for taurine release was determined 6 min after hyposmotic exposure. Data represent the mean maximal rate constants determined from 3 sets of experiments ± SE. *Significantly reduced compared with control (Student's *t*-test). *C–E*: whole cell protein lysates for Western blot analysis were obtained from A2780WT, A2780CisR, and A2780Rev (after 3 and 6 wk) cells exposed to 10 μM Cisplatin for 24 h. Protein expression of LRRC8A, p53, p21, Noxa, Caspase-9, and Bax was determined using specific antibodies (see experimental procedures). β-Actin or Histone 3 were used as loading controls. *C*: representative Western blot. *D*: LRRC8A protein expression relative to β-actin in A2780WT, A2780CisR, and A2780Rev (open bars) control cells and following 24 h Cisplatin exposure (black bars). Data represent 3–7 individual sets of experiments, where ratios are given relative to the untreated WT cells ± SE. * and #: Expression is significantly increased in Cisplatin-treated cell compared with control cells and significantly reduced in Cisplatin-treated A2780CisR cells compared with Cisplatin-treated A2780WT cells, respectively (ANOVA, Fisher LSD method); § indicates expression following cisplatin exposure is significantly increased in A2780 Rev compared with CisR (Student's *t*-test). *E*: graph represents the relative effect of Cisplatin (Cisplatin-treated/respective untreated control) on p53, p21, cleaved Caspase-9, and Noxa protein expression in A2780WT, A2780CisR, and A2780Rev cells. Data represent mean of 3–7 individual experiments ± SE. * and #: Expression is significantly increased in Cisplatin-treated cell compared with control cells and significantly reduced in Cisplatin-treated A2780CisR cells compared with Cisplatin-treated A2780WT cells, respectively (ANOVA, Fisher LSD method). § indicates expression following cisplatin exposure is significantly increased in A2780 Rev compared with CisR (Student's *t*-test).

As development of drug resistance is one of the main issues in chemotherapeutic treatment, we investigated the probability that termination of prolonged Cisplatin treatment, which is normally used to maintain resistance in vitro, could reverse the Cisplatin-resistant phenotype and thereby restore Cisplatin sensitivity in A2780CisR. We have previously shown that Cisplatin resistance in A2780CisR cells correlated with an increased accumulation of taurine due to an increased taurine uptake via the taurine transporter TauT and a concomitant reduction in volume-sensitive taurine release via VSOAC (44). Figure 7, *A* and *B*, shows that abrogation of Cisplatin treatment of A2780CisR for 3 and 6 wk reduces taurine uptake and increases swelling-induced taurine release (shown as the maximal rale constant obtained after osmotic cells swelling) to the same level as observed for A2780WT. From representative Western blots (Fig. 7*C*) and their corresponding quantification (Fig. 7, *D* and *E*), it is seen that interruption of the continued Cisplatin treatment indeed restored *1*) the LRRC8A expression, and *2*) the Cisplatin-induced expression of p53, Noxa and p21^Waf1/Cip1^ as well Cisplatin sensitivity (seen as an increase in cleaved Caspase-9) during the first 3–6 wk. These data demonstrate that cessation of prolonged Cisplatin treatment can restore Cisplatin-induced signaling and sensitivity in A2780 cells.

#### Development of Cisplatin resistance in human alveolar A549 cells involves reduced swelling-induced taurine efflux, reduced LRRC8A expression in the plasma membrane and abolished Caspase-9/-3 activation.

**Fig. 8.**
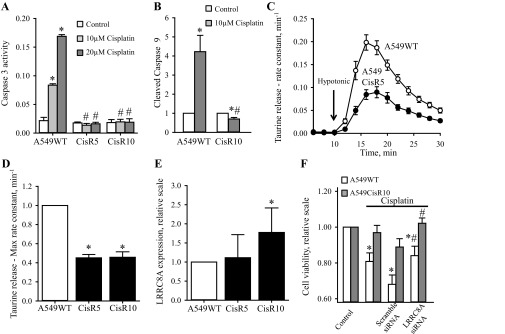
Acquired Cisplatin resistance in human lung A549 cells involves reduction in swelling-induced taurine efflux, increased or unaltered expression of LRRC8A, and abolished Caspase-3/-9 activation. The Cisplatin-resistant A549 cells (A549CisR5 and A549CisR10) were developed by exposing A549WT cells to 5 or 10 μM of Cisplatin, respectively, for a period of 6 mo. To maintain the Cisplatin-resistant phenotype, A549CisR5 and A549CisR10 cells were continuously treated with either 5 μM (A549CisR5) or 10 μM (A549CisR10) Cisplatin once a week. *A*: lysates from A549WT, CisR5, and CisR10 cells exposed to increasing concentrations of Cisplatin (0, 10, and 20 μM, 24 h) were used for selective analysis of Caspase-3 activities. Data represent 3 individual experiments ± SE. * and #: Significantly increased by Cisplatin compared with the respective control and significantly reduced compared with Cisplatin treatment in A549WT cells, respectively (ANOVA, Fisher LSD method). *B*: cleavage of Caspase-9 was determined by Western blot analysis using a specific human antibody against Caspase-9. The Western blot (see Fig. 10) was quantified and illustrated in a bar graph. The effect of Cisplatin treatment (gray bar, 10 µM, 24 h) was set relative to its respective untreated control (open bar). Data represent mean of 4 individual experiment ± SE. * and #: Expression is significantly increased in Cisplatin-treated cell compared with control cells and significantly reduced in Cisplatin-treated A549CisR10 cells compared with Cisplatin-treated A549WT cells, respectively (ANOVA, Fisher LSD method). *C*: A549WT (○) and A549CisR5 (●) cells were loaded before the experiments with [^3^H]taurine, washed, and exposed to isosmotic NaCl medium (320 mOsM) for 10 min and subsequently to hyposmotic medium (200 mOsM) for 20 min (arrow indicates shift in tonicity). Samples were taken every 2 min. The fractional rate constant (min^−1^) for taurine release was determined and plotted vs. time (min). Data represent the mean of 11 sets of experiments. *D*: bar graph represents the maximal rate constant for taurine release in A549WT, CisR5 and CisR10 determined 6 min after hyposmotic exposure (see *A*). Values are given relative to A549WT control and represent the mean of 11 (CisR5) and 5 (CisR10) sets of experiments ± SE. *Significantly reduced compared with A549WT cells (Student's *t*-test). *E*: quantification of whole cell LRRC8A protein levels in A549WT, CisR5, and CisR10 cells determined by Western blot analysis using a monoclonal antibody against human LRRC8A. The LRRC8A expression levels were normalized to β-actin and the ratio given relative to A549WT. Data represent 5 (CisR5) and 10 (CisR10) sets of experiments ± SE. *Significantly increased compared with A549WT cells (Student's *t*-test). *F*: effect of LRRC8A knock-down on cell viability after Cisplatin treatment was determined by MTT (see experimental procedures). A549WT and CisR10 were transfected with either scramble or LRRC8A siRNA; following 1 day transfection, half of the cells were treated with 10 μM Cisplatin and left for another 48 h before measuring cell viability. Data represent 5 individual sets of experiments ± SE. * and #: Significantly decreased by Cisplatin compared with the respective control and significantly increased compared with Cisplatin treatment in scramble siRNA transfected cells, respectively (ANOVA, Fisher LSD method).

**Fig. 9.**
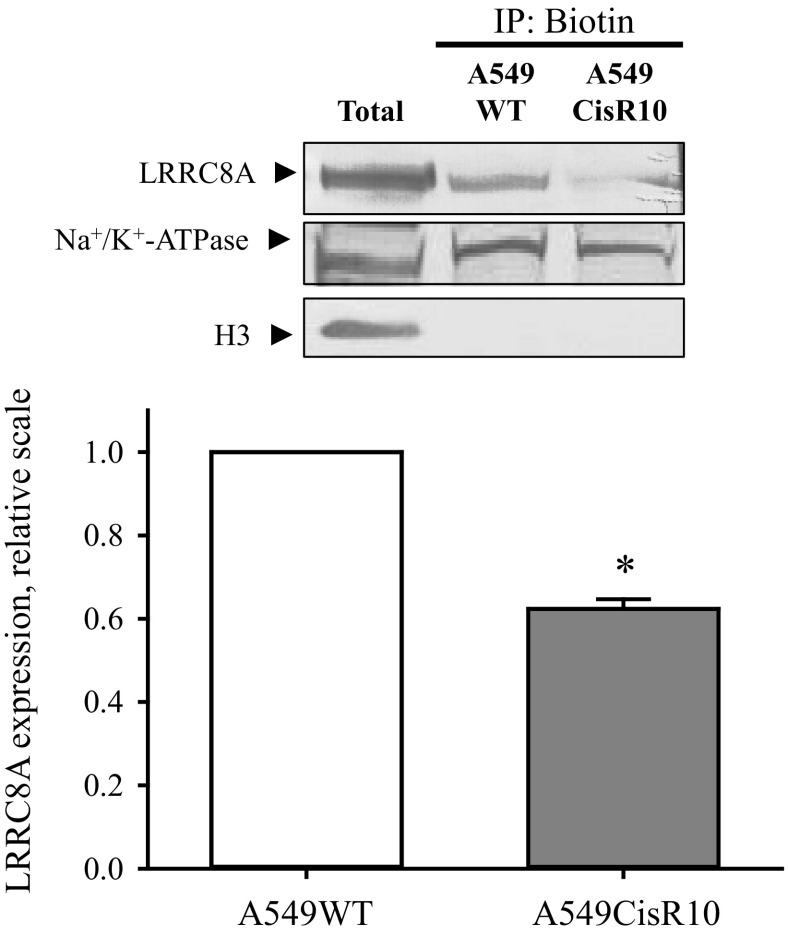
LRRC8A expression in the plasma membrane of wild-type and cisplatin-resistant A549 cells. Proteins exposed to the extracellular compartment in A549WT and A549CisR10 cells were biotinylated, cells lysed, and biotinylated proteins extracted and identified by Western blot as indicated in experimental procedures. *A*: representative blots where histone (H3) was used to verify the absence of intact cells in the preparation and Na^+^/K^+^-ATPase was used as loading control. *B*: protein ratio of LRRC8A/Na^+^/K^+^-ATPase was calculated and given relative to the ratio in A549WT cells. Values are mean of 3 sets of separate experiments. *Significantly reduced compared with A549WT (Student's *t*-test).

As Cisplatin, Oxaliplatin, and Carboplatin are commonly used to treat various types of lung cancer, we wished to establish a Cisplatin-resistant variant of the alveolar A549 adenocarcinoma cell line to test whether development of resistance in this cell type similar to A2780 cells would involve an altered LRRC8A expression, VRAC/VSOAC activity and intracellular signaling along the p53-dependent cascade. Two Cisplatin-resistant A549 cell lines (denoted A549CisR5 and A549CisR10) were developed in our group by exposing A549WT cells to 5 μM or 10 μM Cisplatin for 6 mo. To verify that A549CisR5 and A549CisR10 cell lines are actually resistant to Cisplatin we used the Caspase-3 activity assay and cleavage of Caspase-9, as indicators of the apoptotic progress. Figure 8*A* shows that exposure of A549WT cells to 10 and 20 μM Cisplatin for 24 h results in a 3.6 and 6.8-fold increase in Caspase-3 activation, respectively. However, exposing the A549CisR5 and A549CisR10 cells to the same concentrations of Cisplatin did not cause any Caspase-3 activation, thereby confirming their Cisplatin-resistant nature. Furthermore, Fig. 8*B* shows that exposure of A549CisR10 to 10 μM Cisplatin does not lead to any increase in Caspase-9 cleavage, whereas exposure of A549WT to the same treatment results in a 4-fold increase in Caspase-9 cleavage. From Fig. 8, *C* and *D*, it is seen that A549CisR5 and A549CisR10 show a decreased ability to activate VSOAC and release taurine in response to hyposmolality, i.e., the maximal rate constant in both A549CisR5 and A549CisR10 was reduced to 50% compared with the A549WT cells. To test whether VSOAC activity in A549CisR5 and A549CisR10, similar to findings with the Cisplatin-resistant human ovarian A2780, correlates with a decreased LRRC8A protein expression, we performed Western blot analysis to compare LRRC8A protein expressions in wild-type and resistant A549 cells. Figure 8*E* shows to our surprise that the protein expression of LRRC8A is either unaffected or even increased in the Cisplatin-resistant subtypes compared with A549WT cells. However, similar to our findings with A2780 (Fig. 6*B*) we find cell death in A549WT, induced by 48 h exposure to 10 μM Cisplatin, is reduced, i.e., cell viability increased following transient knock-down of LRRC8A (Fig. 8*F*). Assuming that reduction in the LRRC8A expression/activity results in development of Cisplatin resistance, the increased LRRC8A expression in A549CisR10 cells (Fig. 8*E*) could indicate that the reduced VSOAC activity in the Cisplatin-resistant A549 cells is caused by less LRRC8A being installed in the membrane, failing VSOAC activation, or reduction in the coexpression of other essential VSOAC components (e.g., LRRC8D). To measure expression of LRRC8A in the plasma membrane we isolated biotinylated cell surface proteins from A549WT and A549CisR10 cells and quantified the amount of LRRC8A, Na^+^/K^+^-ATPase (positive plasma membrane control/loading control), and Histone 3 (nuclear control) in both whole cell lysate and purified samples. From Fig. 9 it is seen that even though total LRRC8A expression in A549CisR10 is unaltered or increased compared with A549WT (Fig. 8*E*) significantly less LRRC8A is expressed in the plasma membrane. Hence, the impaired Cisplatin-induced Caspase-9 and -3 activation in A549CisR10 cells seems to reflect impairment of LRRC8A protein expression in the membrane and the concomitant reduction in VSOAC activation. Intracellular trafficking of LRRC8A was not investigated further.

#### Selective knock-down of LRRC8A abolishes Cisplatin-induced expression of p53, MDM2 and p21^Waf1/Cip1^ and reduces p53 activation in alveolar A549 cells.

**Fig. 10.**
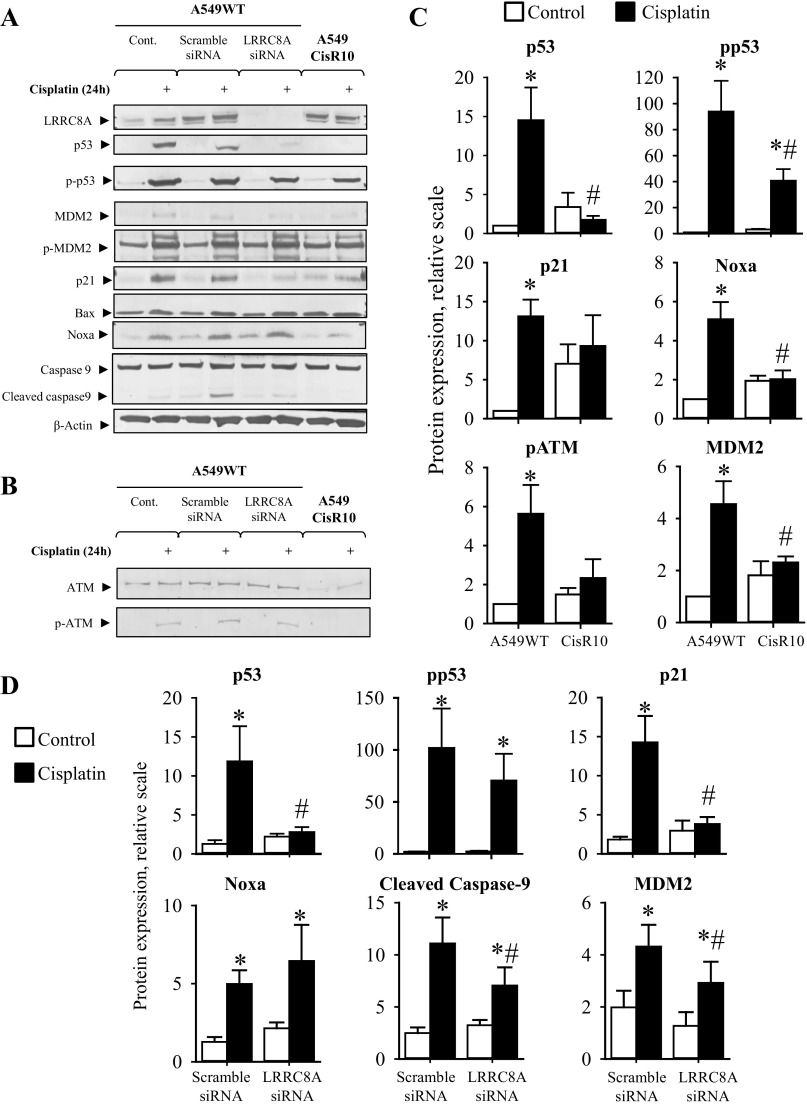
Selective knock-down of LRRC8A abolishes Cisplatin-induced p53, MDM2, and p21^Waf1/Cip1^ expression and reduces Caspase-9 cleavage in wild-type A549 cells. Western blot analysis of whole cell protein lysates obtained from A549WT, A549CisR10 cells and A549WT cells treated with scramble or LRRC8A siRNA exposed to Cisplatin for 24 h. Protein expression of LRRC8A, p53, p-p53 (Ser15), p21, MDM2, p-MDM2 (Ser166), Noxa, Caspase-9 and Bax, as well as ATM and p-ATM (Ser1981) was determined using specific antibodies (see experimental procedures) and β-actin, histone-3, or α-tubulin served as loading controls (only one is shown). *A* and *B*: representative Western blots. *C*: p53, pp53, p21, Noxa, p-ATM, and MDM2 protein expression relative to their loading controls in A549WT and A549CisR10 (open bars) and following 24 h Cisplatin exposure (black bars). Data represent 3 to 6 individual sets of experiments where ratios are given relative to the untreated WT cells ± SE. * and #: Expression is significantly increased in Cisplatin-treated cells compared with respective control cells and significantly reduced in Cisplatin-treated A549CisR10 cells compared with Cisplatin-treated A549WT cells, respectively (ANOVA, Fisher LSD method). *D*: relative protein expression of p53, pp53, p21, Noca, cleaved Caspase-9, and MDM2 in A549WT cell treated with either scramble or LRRC8A siRNA alone (open bars) or in combination with 10 μM Cisplatin (black bars) for 24 h. Data represent 3–6 individual sets of experiments where ratios are given relative to the untreated WT cells ± SE. * and #: Expression is significantly increased by Cisplatin cell compared with respective control cells and significantly reduced in Cisplatin-treated LRRC8A siRNA cells compared with Scramble siRNA cells, respectively (ANOVA, Fisher LSD method).

From Fig. 10, *A–C*, it is seen that 24 h exposure to Cisplatin leads to an increased p53 protein expression and Ser-15 phosphorylation as well as an enhancement of its downstream signaling involving expression of p21^Waf1/Cip1^, MDM2, Noxa, Bax, activation of the ATM kinase, and Caspase-9 cleavage in alveolar A549WT cells. The Cisplatin-induced p53 protein expression and downstream signaling are absent in the corresponding resistant subtype A549CisR10, thus confirming its Cisplatin-resistant phenotype. It is noted that the basal-level of p21^Waf1/Cip1^, Noxa, pATM and MDM2 seems higher in the resistant cells, in a way which was unaffected by Cisplatin treatment (Fig. 10*C*). Several studies indicate that an increase in the expression of antiapoptotic proteins, c-IAP1 and survivin, contribute to chemotherapy resistance (48, 51). Hence, this could explain why cell lines with acquired resistance overcome an increased level of proapoptotic proteins and proteins regulating cell cycle progression. As we have previously shown that administration of the pharmacological VRAC/VSOAC blockers induces Cisplatin resistance in A2780 cells (Fig. 3, *A* and *C*), and that LRRC8A-mediated channel activity is found downregulated or even absent in many Cisplatin-resistant cell lines including A2780CisR, A549CisR and EATC-MDR (see Introduction), we wondered whether knock-down of LRRC8A might directly cause resistance in A549 cells. From Fig. 10*A* (Western blot) and 10*D* (quantification) it is seen that transient knock-down of LRRC8A significantly reduces p53, MDM2 and p21^Waf1/Cip1^ expression, as well as Caspase-9 activation (seen as reduced cleavage) in A549WT cells. The protein expression of LRRC8A, given as the LRRC8A/actin protein band ratio, was in these experiments significantly reduced (*P* < 0.05) from 1.91 ± 0.43 in A549WT cells treated with scramble siRNA to 0.21 ± 0.09 in A549WT cells treated with LRRC8A siRNA for 48 h. The Ser-15 phosphorylation of p53 (Fig. 10*D*) and Ser-166 phosphorylation of MDM2 (0.8 ± 0.5 scrambled siRNA; 1.0 ± 0.6 LRRC8A siRNA; *n* = 3; values relative to control) were not significantly affected. Thus, similar to the findings with anion-channel blockers in A2780 cells, this demonstrates that LRRC8A activity is not important for the phosphorylation of p53, but is entirely essential for Cisplatin-induced stabilization of p53 and its downstream signaling involving increased expression of p21^Waf1/Cip1^.

Cisplatin-mediated DNA damage often leads to a rise in p53 protein stability through ATR/ATM-mediated phosphorylation. We have tested the possibility that LRRC8A directly regulates Cisplatin-induced Ser1981 phosphorylation of ATM, as the phosphorylation was found absent in A549CisR10. From the Western blots shown in Fig. 10*B* it is determined that LRRC8A knock-down does not affect ATM protein expression (1.4 ± 0.3 scrambled siRNA; 1.1 ± 0.2 LRRC8A siRNA; *n* = 3; values relative to control) nor ATM Ser1981 phosphorylation (3.5 ± 0.5 scrambled siRNA; 3.5 ± 1.4 LRRC8A siRNA; *n* = 3; values relative to control). The absent Cisplatin-induced ATM activation in A549CisR10 might reflect a reduced Cisplatin uptake via, e.g., CTR1 or other resistance mechanisms (see Introduction), Hence, LRRC8A is most probably a downstream element to or completely independent of the ATM kinase. This was not investigated further.

#### Selective knock-down of p53 reduces Cisplatin-induced p21^Waf1/Cip1^ and Caspase 9 activation.

**Fig. 11.**
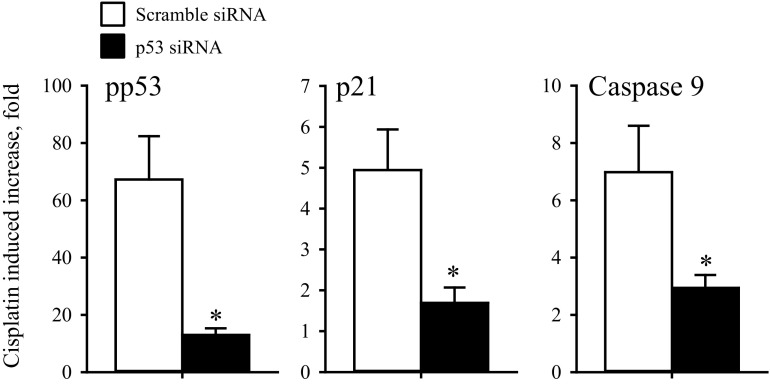
Cisplatin-induced apoptosis/cell cycle arrest in A549 is p53 dependent. Western blot analysis of whole cell protein lysates obtained from A549WT cells treated with scramble siRNA or p53 siRNA. Protein expression of pp53 (Ser15), p21, Caspase-9 and β-Actin/Histone-3 (loading controls) was determined by Western blotting in 4 sets of experiments in cells treated with scramble siRNA or p53 siRNA (48 h) in the presence/absence of Cisplatin treatment (last 24 h, 10 μM). Protein of interest was quantified relative to the loading control, and the Cisplatin-induced increase in protein expression is presented as the protein expression ratio between cisplatin-treated cells and nontreated cells. Data represent mean ratios ± SE from 4 individual sets of experiments. *Expression is significantly reduced by p53 siRNA treatment (Students *t*-test).

It has previously been shown that the apoptotic response of chemoresistant ovarian cancer cells is significantly enhanced following reconstitution of p53 (21). We have now investigated whether Cisplatin-induced apoptosis and cell cycle arrest are both dependent on p53. Comparing p53 siRNA transfected A2780WT cells with scramble siRNA transfected cells we find in two independent experiments that p53 silencing reduces the increase in p53 and p21^Waf1/Cip1^ protein expression and Caspase 9 activity following 24 h exposure to Cisplatin (10 μM) from 56- to 4-fold (p53), 24- to 12-fold (p21^Waf1/Cip1^), and 29- to 4-fold (Caspase 9). Similarly, we find in 4 experiments that p53 silencing in A549 cells reduces pp53 and p21^Waf1/Cip1^ protein expression and Caspase 9 activity following Cisplatin exposure, significantly from 67 ± 15- to 13 ± 2-fold (pp53), 5 ± 1- to 1.7 ± 0.4-fold (p21^Waf1/Cip1^), and 7.0 ± 1.6- to 2.9 ± 0.5-fold (Caspase 9) (Fig. 11). Hence, Cisplatin-induced cell cycle arrest and apoptosis in wild-type A2780 and A549 cells are p53 dependent.

## DISCUSSION

Development of drug resistance is a continuing issue in chemotherapeutic treatment. As indicated in the introduction, the mechanism of platinum (II) resistance involves direct changes in drug transport systems resulting in a reduced intracellular drug accumulation, an enhanced drug detoxification and damage tolerance, as well as changes in the apoptotic signal transduction pathways (12, 24, 25, 32, 46). During the past 15 years, it has however turned out that a hallmark in apoptosis is isosmotic cell shrinkage, a process that is termed apoptotic volume decrease (AVD) (29, 37).

The involvement of ion channels and transporters has been associated with cancer progression and development of chemotherapeutic resistance for a long time. Back in 1992, Gollapudi and coworkers (13) recognized an abnormal chloride conductance in a multidrug-resistant human promyelogenous HL60/AR leukemia cell line. The baseline Cl^−^ currents (+100 mV) were consistently lower in HL60/AR compared with the drug-sensitive HL60 parental cells. Furthermore, blocking Cl^−^ channel activity in drug-sensitive HL60 cells with DIDS resulted in deceased intracellular accumulation of the chemotherapeutic drug daunorubicin and increased drug resistance, thus pointing to the role of VRAC/VSOAC in development of MDR.

In multidrug-resistant Ehrlich ascites tumor cells (MDR-EATC), it has likewise been demonstrated that acquired drug resistance involves a reduced activity of VRAC/VSOAC mediating the volume-sensitive Cl^−^ currents and efflux of amino acids, e.g., taurine (37, 38). Administration of the pharmacological VRAC/VSOAC inhibitors, e.g., DIDS/NS3728 and the TASK-2 cation channel blocker clofilium, have been shown to reduce Cisplatin-induced cell shrinkage (AVD_1_) as well as both Cisplatin- and anoxia-induced Caspase-3 activation in drug-sensitive EATC-WT and A549WT (19, 37). In accordance, we find that both VRAC/VSOAC blockage (NS3728 and DIDS) and transient knock-down of LRRC8A reduces the Cisplatin-induced Caspase-3 activation and increases cell viability in A2780WT to the same level as observed in A2780CisR (Figs. 5 and 6).

**Fig. 12.**
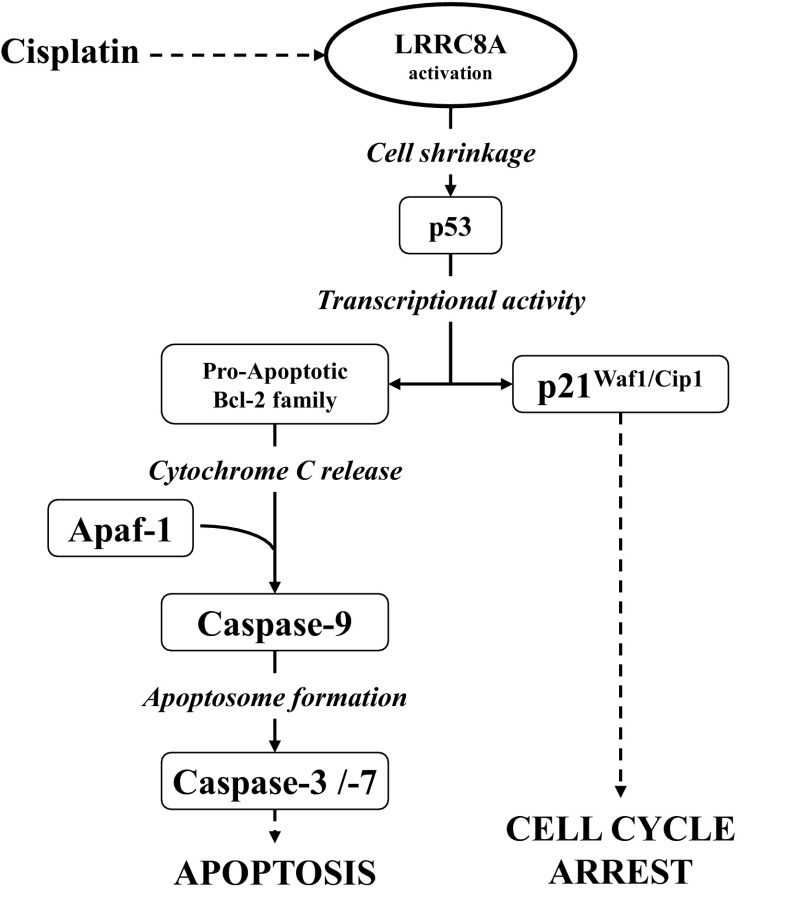
Role of LRRC8A in cell signaling to apoptosis/cell cycle arrest via the intrinsic, volume sensory, and extrinsic apoptotic pathway. Induction of apoptosis and cell cycle arrest via the intrinsic cell death pathway is generally elicited by cellular stress as DNA damage (adducts and cross-links). Induction of apoptosis by Cisplatin involves activation of the kinases ATM/ATR (not shown) and activation/increased protein expression of LRRC8A. The dashed line between DNA damage and LRRC8A activation indicates that the precise signaling event is unknown. Activation of LRRC8A causes cell shrinkage (apoptotic volume decrease), which directly signals to the transcription factor p53. Increased stability and activation of p53 increase the gene expression of both p21 and Bax. Increased expression of p21 leads to G1/S and G2/M cell cycle-arrest through blockage of cyclin-dependent kinase 1 and 2 (CDK-1 and -2), and increased expression of Bax leads to increased mitochondrial outer-membrane permeabilization, cytochrome c release, activation of Caspase-9, and thus activation of executer caspases (e.g., Caspase-3 and -7).

As described previously, one of the major players in Cisplatin-induced apoptosis is the transcription factor p53. We find that p53 siRNA transfection reduces Cisplatin-induced Caspase-9 activation indicating that Cisplatin-induced apoptosis in both WT A2780 and A549 cells is p53-dependent. Genome sequencing has revealed that over 50% of all human malignancies exhibit p53 point mutations located in the DBD region (35). Many of these mutations lead to its miss-folding, decreased DNA binding capability, and even a gain-of-function of p53. In many other tumor cells p53, although it was intact, was found to be inactive due to an enhanced degradation and less activation. Acquired p53 inactivation or gain-of-functions mutations are often associated with aggressive tumor growth, development of chemotherapeutic resistance, and poor survival prognosis (35). Compton and coworkers (7) have in addition demonstrated that mitochondrial dysfunction seems to protect cells from γIR-induced cell death by repression of p53 activity. Recent studies clearly identify that acute hyperosmotic stress leads to a biphasic stabilization and activation of the transcription factor p53 in mouse NIH3T3 fibroblasts, supposedly through Ser-15 phosphorylation by the serine/threonine kinase ATM and the p38 MAP kinase (27). This could indicate that LRRC8A is a mitochondrial protein, i.e., that lack of LRRC8A in the mitochondrial membrane leads to mitochondrial dysfunction and repression of p53 activity. LRRC8A is highly expressed in the plasma membrane (50) and we therefore assume that cell shrinkage regulates p53 either independently of the mitochondria or that shift in cell volume contributes to mitochondrial dysfunction and hence p53 regulation. In our studies, we clearly demonstrate that the VRAC/VSOAC compound LRRC8A is essential for Cisplatin-induced increase in p53, MDM2, and p21^Waf1/Cip1^ protein level as well as Caspase-9 and -3 activation in both human ovarian A2780 and alveolar A549 cells. In contrast, LRRC8A inhibition had no effect on hyperosmotic stress-induced Caspase-3 activity and only a slight effect on TNFα-induced Caspase-3 activation in A2780, thus indicating that LRRC8A acts upstream to shrinkage-induced and to TNFα-induced apoptosis. We furthermore find that transient expression of LRRC8A in A2780CisR reestablishes the sensitivity toward Cisplatin (Fig. 6) and that cessation of a prolonged Cisplatin treatment, which is used to maintain resistance in vitro, reverses the Cisplatin-resistant phenotype and restore Cisplatin sensitivity. This confirms that the acquired Cisplatin resistance in A2780 cells is a reversible condition. In drug-sensitive human K562 erythroleukemia cells and RK562 cells, which are doxorubicin resistant and express P-glycoprotein and multidrug resistance proteins (MDR1 and MRP1), Xu and colleagues (54) have reported that administration of the board-spectrum anion-channel blocker 5-nitro-2-(3-phenylpropylamino)benzoic acid (NPPB) significantly blocks Cisplatin-induced activity and apoptosis in both K562 and RK562 cells. Moreover, they found that NPPB reduced the Cisplatin-induced protein expression of Bax/Bcl2, cytochrome c and Caspase-3, thus supporting that LRRC8A-mediated VRAC/VSOAC channel activity is important for Cisplatin cytotoxicity and the intrinsic apoptotic pathway. A schematic representation of the intrinsic apoptotic pathways and the involvement of LRRC8A is given in Fig. 12. It is seen that LRRC8A is essential for the AVD response which is an initial step in the intrinsic pathway. Xu and colleagues (54) also show that Cisplatin treatment reduces the mRNA accumulation of cyclin D1 and the chloride channel 3 (ClC-3) in both K562 and RK562 cells in a way avoided by cotreatment with NPPB. The same group (47) later reported that ClC-3 is an anti-apoptotic channel, as selective knock-down of ClC-3 expression caused inhibition of Akt and autophagy, and enhanced Cisplatin toxicity in human malignant U251 glioma cell line. Thus not all anion channels are considered to be proapoptotic like VRAC/VSOAC.

We have previously demonstrated that acute exposure of Cisplatin-sensitive A2780 cells to 5 and 10 μM Cisplatin for 18 h results in a marked increase in LRRC8A protein content, whereas the same treatment had no effect on the LRRC8A protein expression in A2780CisR (44). Similar to this, studies by Planells-Cases and coworkers (36) indicate that Cisplatin-induced taurine release is abolished in LRRC8A knock-out HEK cells, and they define LRRC8A and LRRC8D as essential for both swelling-induced taurine release as well as development of drug resistance. As indicated in the Introduction, sequence analysis revealed that the LRRC8 family are considered to originate from a common ancestor and to be closely related to pannexins. Pannexins, like the LRRC8 membrane-spanning proteins, form hexameric channels and are known to be involved in leakage of Ca^2+^ from the ER and ATP-dependent cell death (1). As an example, Pannexin 1 channels mediate “find-me” signals (e.g., ATP release) and increase membrane permeability during Fas-receptor stimulated apoptosis (6). The pannexin family is structurally related to connexins, which also form hexameric complexes known as connexons or connexin hemichannels. These comprise gap junctions that connect the cytosol of neighboring cells, thus allowing bidirectional flow of ions and signaling molecules such as amino acids and nucleotides (2). Interestingly, like for LRRC8A, connexin 43 (Cx43) protein expressions are report to be downregulated in Cisplatin-resistant lung A549/DDP cancer cells compared with Cisplatin-sensitive A549 cells (56). In addition, overexpression of Cx43 enhanced Cisplatin cytotoxicity in mesothelioma H28 cells (41). The A549/CDDP cells were found to acquire an epithelial-mesenchymal transition (EMT) phenotype, with morphological changes including acquirement of a spindle-like fibroblastic phenotype, downregulation of E-cadherin, upregulation of mesenchymal markers (e.g., vimentin, Snail and Slug), and increased capability of migration and invasion (56). Yu and coworkers (56) found that overexpression of Cx43 reversed EMT and Cisplatin resistance in the A549/CDDP cells. Mesenchymal integrins are often highly overexpressed in tumor cells (9), and we have previously suggested that the Cisplatin-resistant phenotype in Ehrlich ascites Lettré cells is acquired through changes in members of the integrin family, involving upregulation of integrins often seen in mesenchymal cells (43). Inhibition of Cx43 with the gap-junction inhibitor αGA leads to enhanced drug resistance in a resistant version of the A2780 cells (ACRP) (31). Paradoxically, Li and coworkers (31) also find that the protein expression of Cx43 is markedly upregulated in ACRP compared with A2780. In our Cisplatin-resistant A549 cells, we find that even though the total LRRC8A protein expression is elevated compared with A549WT, the amount of LRRC8A in their plasma membrane is significantly reduced which could explain the concomitant 50% reduction in VRAC/VSOAC activity. This indicates that changes in the overall protein contents of certain genes do not necessarily equalize to the same alteration in activity.

In conclusion, we have shown that the VRAC/VSOAC component LRRC8A is an essential regulator of Cisplatin-induced p53 protein activity and its downstream signaling involving increased expression of p21^Waf1/Cip1^ and MDM2, as well as activation of Caspase-9 and -3. Our data clearly illustrate that downregulation/reduced translocation to the plasma membrane and activation of LRRC8A contribute to development of resistance against Cisplatin-induced apoptosis in ovarian and lung carcinoma cells. Activation of LRRC8A containing channels is upstream to apoptotic volume decrease as hypertonic cell shrinkage induces apoptosis independent of the presence of LRRC8A.

## GRANTS

The present work was supported by “Læge Sofus Carl Emil Friis og hustru Olga Doris Friis' legat,” “Fabrikant Einar Willumsens Mindelegat,” “Familien Erichsens Mindefond,” and The Carlsberg Foundation.

## DISCLOSURES

No conflicts of interest, financial or otherwise, are declared by the author(s).

## AUTHOR CONTRIBUTIONS

Author contributions: B.H.S., E.K.H., and I.H.L. conception and design of research; B.H.S., D.N., U.A.T., and I.H.L. performed experiments; B.H.S., D.N., U.A.T., E.K.H., and I.H.L. analyzed data; B.H.S., D.N., U.A.T., E.K.H., and I.H.L. interpreted results of experiments; B.H.S. and I.H.L. prepared figures; B.H.S. and I.H.L. drafted manuscript; B.H.S., U.A.T., E.K.H., and I.H.L. edited and revised manuscript; B.H.S., D.N., U.A.T., E.K.H., and I.H.L. approved final version of manuscript.
